# Supplementary material for: Risk Factors of Infection in Relapsed/Refractory Multiple Myeloma Patients Treated with Lenalidomide and Dexamethasone (Rd) Regimen: Real-Life Results of a Large Single-Center Study
Source: J Clin Med. 2022 Oct 7;11(19):5908. doi: 10.3390/jcm11195908 (PMC9572774; doi:10.3390/jcm11195908)
Supplement: Supplementary file 1 [file jcm-11-05908-s001.zip › jcm-1918975-supplementary.pdf]

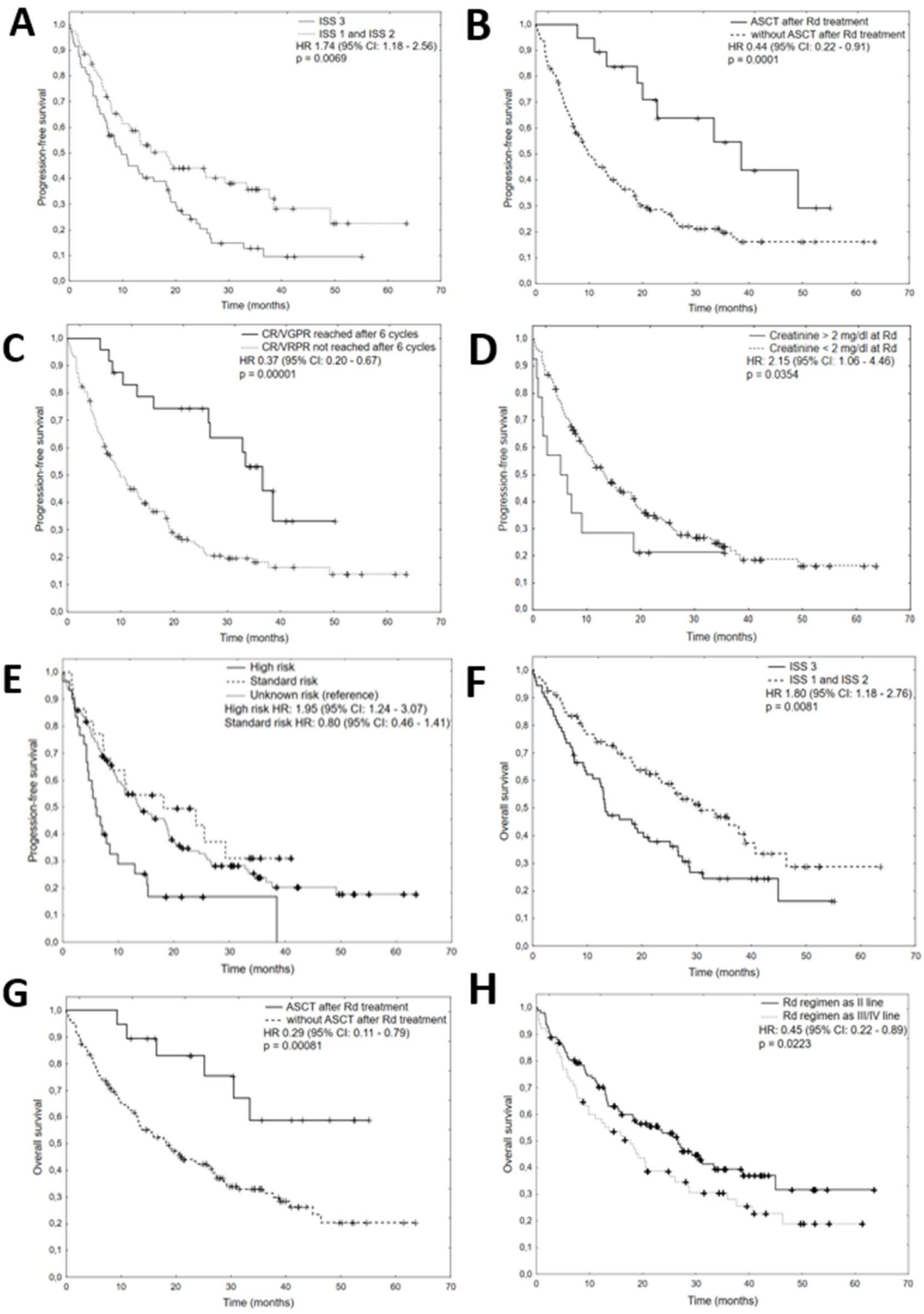

Figure S1. Kaplan Meier plots for independent factors influencing PFS (A- ISS 3, B- AHSCT after Rd, C- CR/VGPR after cycle 6 of Rd, D- creatinine >2 mg/dl, E- high-risk cytogenetic group according to IMWG) and OS (F- ISS 3, G- AHSCT after Rd, H- Rd regimen in II line of treatment).
